# Tyrosine Kinase Inhibitors for the Treatment of Chronic-Phase Chronic Myeloid Leukemia: Long-Term Patient Care and Management

**DOI:** 10.6004/jadpro.2016.7.1.3

**Published:** 2016-01-01

**Authors:** Stephanie Bauer,1, Susan Buchanan,2, Irene Ryan,3

**Affiliations:** 1Department of Internal Medicine, Washington University, St. Louis, Missouri; 2Adult Leukemia Program, Dana-Farber Cancer Institute, Boston, Massachusetts; 3Department of Internal Medicine and Department of Hematology/Oncology, University of Michigan Health System, Ann Arbor, Michigan

## Abstract

Several tyrosine kinase inhibitors (TKIs) are now approved for the treatment of chronic myeloid leukemia in chronic phase. The efficacy of these drugs has been repeatedly demonstrated, as has their tolerability in most patients. However, late and chronic toxicities become an important issue for many patients facing long-term TKI exposure. For patients on long-term imatinib, gastrointestinal events, fluid retention, muscle cramps, fatigue, and hepatotoxicity are among the most common and most clinically relevant adverse events (AEs). A few of these have also emerged as important AEs with some of the newer TKIs. Distinct long-term toxicity concerns have emerged for dasatinib (pleural effusion, pulmonary hypertension, headache, and dyspnea) and nilotinib (rash, headache, myalgia, alopecia, and hyperglycemia), whereas due to the recent approval of bosutinib and ponatinib, their long-term toxicity profiles have not been fully characterized. Clinical experience with each of these drugs is accumulating, and ensuring proper adherence and monitoring for potential AEs is essential for effective treatment.

The availability of multiple BCR-ABL tyrosine kinase inhibitors (TKIs) presents health-care professionals (HCPs) with an important decision when assigning therapy for patients with chronic-phase chronic myeloid leukemia (CML-CP; Kantarjian, Baccarani, Jabbour, Saglio, & Cortes, 2011). Health-care professionals must decide between the approved TKIs by weighing therapeutic efficacy, convenience, the patient’s relevant comorbidities, and patient and HCP preferences, among other considerations.

As TKI therapy is typically administered continuously for patients with CML, the long-term clinical management of TKI-related adverse events (AEs) is an important task for HCPs. A recent study that monitored patients with CML through 1 year of treatment found that approximately one-third of patients experienced persistent moderate to severe symptoms, most commonly fatigue, drowsiness, disturbed sleep, muscle soreness, cramping, and memory deficit. For many patients, these symptoms interfered with day-to-day functioning (Williams et al., 2013). In the future, increased coordination between several specialists (such as primary care physicians, cardiologists, and endocrinologists) may become necessary when caring for patients on long-term TKI therapy (Thanopoulou & Judson, 2012).

Although more than 10 years of safety data are available for imatinib, dasatinib (Sprycel) and nilotinib (Tasigna) have been available in the front-line setting for approximately 4 years, and bosutinib (Bosulif) and ponatinib (Iclusig) have been used in the second-line and salvage settings for an even shorter period of time. Here we review significant AEs and other relevant considerations associated with BCR-ABL TKIs approved for patients with CML, with an emphasis on practical long-term clinical management.

## BCR-ABL TKIS APPROVED IN CML-CP

Imatinib was the first BCR-ABL TKI to obtain clinical approval from the US Food and Drug Administration (FDA) for the treatment of Philadelphia chromosome–positive (Ph+) CML (Druker et al., 2001, 2006; Novartis Pharmaceuticals, 2014a), and several newer BCR-ABL TKIs have been approved in recent years. Both dasatinib and nilotinib are approved for front-line therapy of CML-CP based on their superior efficacy vs imatinib in phase III clinical trials (Kantarjian et al., 2010; Saglio et al., 2010a). Dasatinib, nilotinib, and bosutinib are all approved for the treatment of patients who are resistant to or intolerant of prior therapy, and ponatinib is approved for patients with the *T315I BCR-ABL* mutation and those for whom no other TKI is indicated (Ariad Pharmaceuticals, 2014; Bristol-Myers Squibb Company, 2014; Novartis Pharmaceuticals, 2014a, 2014b; Pfizer, 2013a). Each of the approved TKIs displays distinct clinical activity, including different AE profiles (Table 1).

**Table 1:**
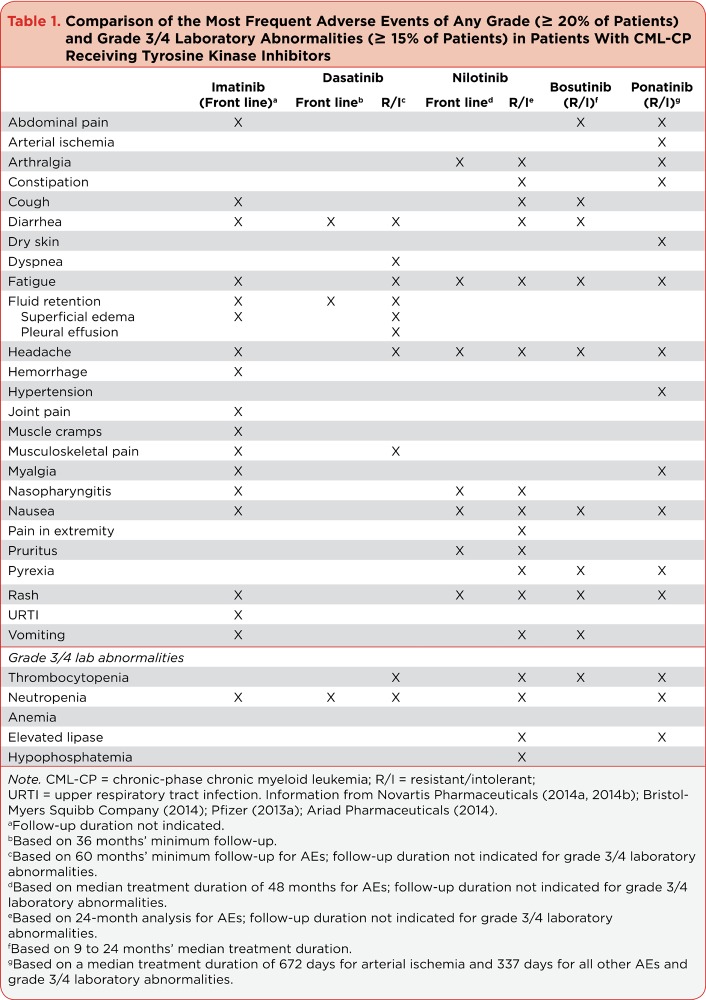
Comparison of the Most Frequent Adverse Events of Any Grade (≥ 20% of Patients) and Grade 3/4 Laboratory Abnormalities (≥ 15% of Patients) in Patients With CML-CP Receiving Tyrosine Kinase Inhibitors

**Imatinib**

Imatinib, first approved by the FDA in 2001, is indicated for the treatment of adult and pediatric patients with newly diagnosed CML-CP as well as patients with CML in any phase (CP, accelerated phase [AP], or blast crisis [BC]) following failure of interferon á. The recommended dose of imatinib for adults with CML-CP is 400 mg once daily. Imatinib has no contraindications or boxed warnings (Novartis Pharmaceuticals, 2014a).

In addition to the pivotal International Randomized Study of Interferon and STI571 (IRIS) trial (Hochhaus et al., 2009; O’Brien et al., 2003), on the basis of which imatinib received its FDA approval, imatinib has been used as the comparator arm in phase III trials of dasatinib (Kantarjian et al., 2010; Kantarjian et al., 2012), nilotinib (Larson et al., 2012; Saglio et al., 2010a), and bosutinib (Cortes et al., 2012). In each of these trials, imatinib was generally well tolerated. However, many patients experienced some mild to moderate toxicity (O’Brien et al., 2003). Gastrointestinal (GI) events (including nausea, vomiting, and diarrhea), fluid retention, muscle cramps, fatigue, and hepatotoxicity were among the most common AEs in patients on imatinib. With 19 months’ median follow-up in IRIS, 43.7%, 32.8%, and 16.9% of patients in the imatinib arm experienced nausea, diarrhea, and vomiting, respectively; 55.5% experienced superficial edema; 38.3% muscle cramps; and 34.5% fatigue (O’Brien et al., 2003). The safety profile was similar after 5 years of follow-up, with no new safety signals reported after 8 years (Deininger et al., 2009; Druker et al., 2006). These AEs have emerged as particularly relevant in daily practice, with low-grade events sometimes becoming chronic complaints of patients receiving long-term imatinib therapy.

In a health-related quality-of-life survey involving 448 patients with CML who had been on imatinib for a median of 5 years, symptoms included edema (69.6%, of which 25.4% were reported as occurring "quite a bit/very much"), musculoskeletal pain (72.6%, frequent in 25.3%), muscle cramps (77.9%, frequent in 30.5%), and fatigue (82.1%, frequent in 29.3%). Gastrointestinal symptoms also occurred and included diarrhea (43.2%, frequent in 15.1%) and nausea (28.2%, frequent in 4.8%; Efficace et al., 2011).

These AEs, including chronic events, can be effectively managed. Gastrointestinal symptoms are often relieved when imatinib is taken with food and water (Deininger, O’Brien, Ford, & Druker, 2003; National Comprehensive Cancer Network [NCCN], 2014). A food diary can help identify which foods (often lactose/dairy products) may be contributing to the GI disturbance(s). For immediate management of GI toxicity, antinausea and antidiarrheal medications such as loperamide with or without diphenoxylate/atropine can be used (Deininger et al., 2003). The 2014 NCCN CML guidelines recommend supportive care, diuretics, and dose interruption, reduction, or discontinuation for patients with fluid retention on imatinib and suggest performing an echocardiogram to check left ventricular ejection fraction (NCCN, 2014). Periorbital edema often responds to topical phenylephrine 0.25% (Deininger et al., 2003). For patients with severe fatigue on imatinib, methylphenidate or modafinil is often used for managing cancer-related fatigue (Escalante & Manzullo, 2009) and may provide relief.

Comorbid conditions such as hypertension, diabetes mellitus, and obesity can also contribute to fatigue and muscle cramps; optimal management of these conditions should be emphasized. In addition, patients can improve both their physical and emotional well-being by increasing their overall activity level.

The imatinib label also lists cardiac, liver, and kidney toxicities and immunosuppression as potential concerns for patients with long-term exposure (Novartis Pharmaceuticals, 2014a). Although hepatotoxicity most often occurs early during treatment (Deininger et al., 2003), transaminase, bilirubin, and alkaline phosphatase levels should be routinely monitored while patients remain on imatinib. Liver function tests should be performed monthly for the first 2 months and then every 3 months; for patients with abnormal test results, imatinib dosing should be interrupted and/or reduced according to instructions in the label.

Severe congestive heart failure and left ventricular dysfunction have occurred in patients on imatinib (Novartis Pharmaceuticals, 2014a); however, patients with cardiac events may have relevant risk factors and comorbidities at baseline (Giles et al., 2013; Novartis Pharmaceuticals, 2014a), highlighting the importance of considering each patient’s medical history and managing comorbidities and risk factors appropriately.

**Dasatinib**

Dasatinib was initially approved by the FDA in 2006 and is indicated for patients newly diagnosed with CML-CP and patients with CML in any phase who are resistant to or intolerant of prior therapy, including imatinib (Bristol-Myers Squibb Company, 2014). For patients with CML-CP, the recommended dose is 100 mg once daily. Dasatinib has no contraindications or boxed warnings.

Of the AEs reported with dasatinib use, pleural effusions, headaches, fatigue, and dyspnea are particularly relevant in daily practice. Fluid retention was reported most commonly among patients receiving front-line dasatinib in the pivotal phase III Dasatinib Versus Imatinib Study in Treatment-Naive CML Patients (DASISION) trial, with a reported frequency of 31% (any grade; grade 3/4, 3%) after 3 years’ minimum follow-up; 19% of patients had pleural effusions (any grade; grade 3/4, 2%; Jabbour et al., 2014). Notably, pleural effusions developed despite protocol-mandated prospective chest x-rays at baseline, after 6 months on treatment, and as clinically indicated (Kantarjian et al., 2012). This frequency is also consistent with that of pleural effusions on second-line dasatinib (reported in 25% of patients [any grade; grade 3/4, 5%] after 6 years of follow-up; Shah et al., 2012). Overall, the rate of new pleural effusions (any grade) in patients on front-line dasatinib increased by approximately 4% to 5% each year (Jabbour et al., 2014; Kantarjian et al., 2010, 2012).

Headaches and fatigue were less common with front-line dasatinib (13% and 9% of patients, respectively, by 3 years’ minimum follow-up) but have emerged as clinically relevant. Additionally, although dyspnea was infrequent in DASISION, occurring in < 10% of patients by 3 years (Jabbour et al., 2014), it was among the most common AEs in patients receiving second-line dasatinib (reported in 30% of patients [any grade; grade 3/4, 5%] after 15.2 months’ median follow-up; Hochhaus et al., 2008).

Dasatinib treatment should be suspended for patients with pleural effusions, which can arise early or late after initiation of therapy (Jabbour et al., 2014; Kantarjian et al., 2010; Porkka et al., 2010). Temporary dose interruptions, diuretics, and pulse corticosteroids are often effective for pleural effusions (Shah et al., 2008). If symptoms resolve, dasatinib can be reinitiated at a reduced dose (NCCN, 2014). Patients with dyspnea must be evaluated for effusions and other contributing factors; chest x-ray and echocardiogram may help to identify the cause.

Pulmonary arterial hypertension (PAH) is a rare but serious dasatinib toxicity with the potential of occurring at any time after treatment is initiated (Montani et al., 2012; NCCN, 2014; Tatarczuch, Seymour, Creati, Januszewicz, & Burbury, 2013). The 2014 NCCN guidelines recommend evaluating patients for underlying cardiopulmonary disease (as no specific methodology is given, evaluation must be done per provider discretion) prior to and during dasatinib therapy and discontinuing dasatinib permanently in any patient with PAH (NCCN, 2014).

**Nilotinib**

Nilotinib was initially approved by the FDA in 2007 and is indicated at a dose of 300 mg twice daily for newly diagnosed adults with CML-CP and 400 mg twice daily for patients with CML-CP or CML-AP who are resistant to or intolerant of previous therapy, including imatinib. The nilotinib label includes a boxed warning for QT-interval prolongation and sudden death; hypomagnesemia, hypokalemia, and long QT interval are listed as contraindications for nilotinib treatment (Novartis Pharmaceuticals, 2014b).

With 1-year minimum follow-up in the pivotal Evaluating Nilotinib Efficacy and Safety in Clinical Trials—Newly Diagnosed Patients (ENESTnd) study, AEs occurring in patients on the nilotinib 300 mg twice-daily arm included rash (31%), pruritus (15%), headache (14%), fatigue (11%), nausea (11%), myalgia (10%), alopecia (8%), and diarrhea (8%). Notably, the majority of these events were grade 1/2. Hyperglycemia was observed in 36% of patients (6% grade 3/4; Saglio et al., 2010a). The safety profile with nilotinib was similar with longer follow-up (Larson et al., 2014). Adverse events observed in clinical practice include rash (often pruritic), headache, myalgia, alopecia (especially in women), and hyperglycemia. Among patients in ENESTnd, low-grade chronic AEs that significantly reduced patient quality of life after 4 years of follow-up included GI events, fatigue, peripheral edema, muscle spasm, arthralgia, insomnia, and anxiety. Notably, these were less frequent with nilotinib than with imatinib (Chen et al., 2013).

Management of certain AEs on nilotinib may be similar to that with other TKIs. As with dasatinib, headaches are most common shortly after drug initiation and often resolve spontaneously with time. Aspirin/acetaminophen/caffeine or other nonsteroidal anti-inflammatory drugs may provide short-term relief; narcotics, triptans, or TKI dose reductions may also be considered. Myalgia usually resolves spontaneously with time; stretching, walking, or taking acetaminophen can help relieve symptoms. Rashes may abate with time or following dose interruption or reduction.

Although uncommon (occurring in < 1% of patients), thyroid abnormalities have been observed with nilotinib (Kim et al., 2010; Novartis Pharmaceuticals, 2014b). Pancreatitis can be a common AE (occurring in ≥ 1% and < 10% of patients), and lipase levels should be evaluated at baseline and monthly or as considered appropriate (Novartis Pharmaceuticals, 2014b). When observed, hyperglycemia is usually mild (Rea et al., 2012). Most cases are easily controlled with single-agent sulfonylurea or do not require medical intervention. Few patients require nilotinib dose reduction or interruption due to hyperglycemia (Nicolini et al., 2012), and most patients with diabetes do not have changes to glycosylated hemoglobin or require modifications to their diabetic therapy (Saglio et al., 2010b). However, care must be taken if HCPs consider administering metformin in patients on nilotinib because each of these drugs has been shown to induce hepatotoxicity in a minority of patients (Larson et al., 2012; Miralles-Linares et al., 2012).

Atherosclerotic cardiovascular events, including peripheral arterial occlusive disease (PAOD), have occurred on nilotinib. By 5 years of follow-up in ENESTnd, 7 of 282 (2.5%), 7 of 281 (2.5%), and 0 of 283 patients in the nilotinib 300 mg twice-daily, nilotinib 400 mg twice-daily, and imatinib 400 mg once-daily arms, respectively, experienced PAOD events (Larson et al., 2014). A retrospective cohort analysis of 2,390 patients with CML-CP treated with front-line nilotinib, imatinib, or no TKI therapy found a cumulative PAOD incidence rate per 100 patient-years of 0.5% in the nilotinib cohort, 0.1% in the imatinib cohort, and 0.6% in the no-TKI cohort. Notably, 11 of the 12 patients with PAOD in the 3 cohorts had known risk factors at baseline (Giles et al., 2013).

Major cardiovascular risk factors include, among others, smoking, hypertension, elevated levels of total serum or low-density lipoprotein cholesterol, low levels of high-density lipoprotein cholesterol, and diabetes (Grundy et al., 1999) and should be managed in all patients with CML per current recommendations (Eckel et al., 2013).

The 2014 NCCN guidelines list PAOD as a rare but serious nilotinib toxicity; patients with risk factors should be monitored very closely, and nilotinib should be discontinued permanently if PAOD occurs (NCCN, 2014). In addition to the boxed warning for QT prolongation and sudden death on nilotinib, patients must be monitored periodically for both hypokalemia and hypomagnesemia; nilotinib should not be administered if any of these conditions is detected. Electrolyte abnormalities must be evaluated and corrected prior to beginning nilotinib; electrolyte evaluation should be repeated 1 week after therapy is initiated. Once normalized, electrolyte levels should be monitored throughout treatment.

Concomitant administration of strong cytochrome P450 3A4 (CYP3A4) inhibitors or other drugs that can cause QT prolongation (Table 2) should be avoided. It is also valuable to perform an electrocardiogram at baseline and after 1 week of treatment. In addition, because nilotinib bioavailability can increase by up to 82% when given 30 minutes after a high-fat meal (vs the fasted state), nilotinib must be taken on an empty stomach; no food should be consumed 2 hours before and 1 hour after a dose (Novartis Pharmaceuticals, 2014b).

**Table 2:**
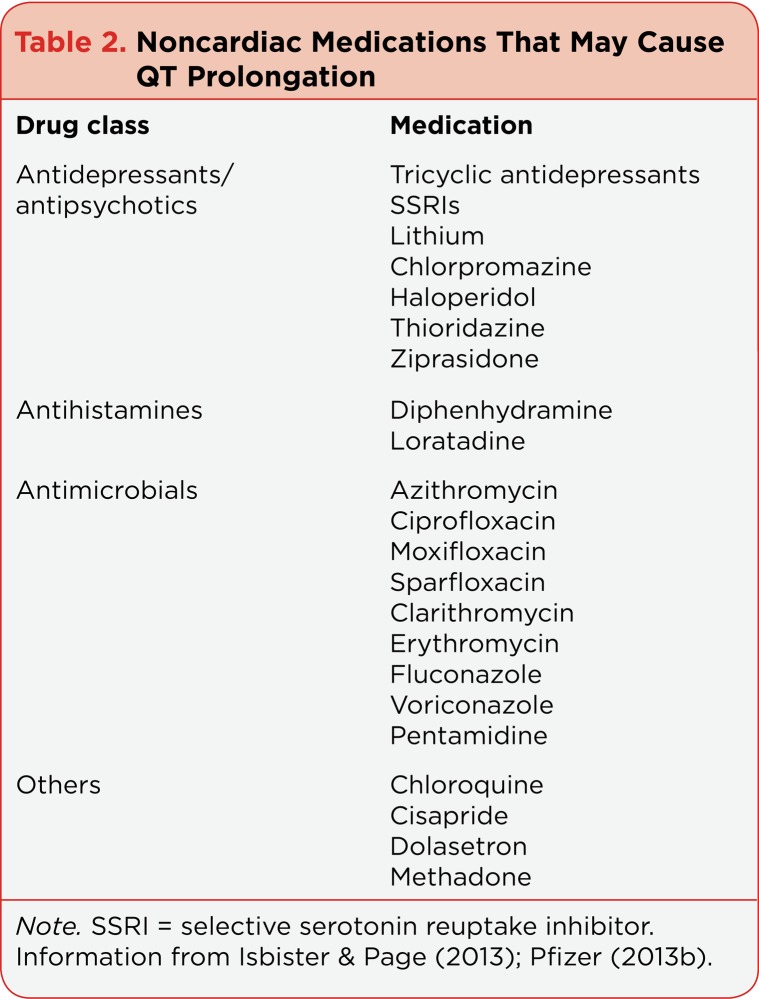
Noncardiac Medications That May Cause QT Prolongation

**Bosutinib**

Bosutinib was initially approved by the FDA in 2012 and is indicated for adult patients with CML in any phase who are resistant to or intolerant of prior therapy. The recommended dose of bosutinib is 500 mg once daily. Patients who do not achieve complete hematologic response by week 8 or complete cytogenetic response by week 12 may consider dose escalation to 600 mg daily if they have not had grade ≥ 3 AEs. Bosutinib does not have any boxed warnings and is contraindicated only in patients with hypersensitivity to bosutinib (Pfizer, 2013a).

Diarrhea, the most common AE seen with bosutinib, is an important consideration in daily practice. In the pivotal phase II trial of second-line bosutinib, 84% of patients had experienced diarrhea (any grade; grade 3/4 in 9%) by 24.2 months’ median follow-up (Cortes et al., 2011). By 12 months’ minimum follow-up in the phase III Bosutinib Efficacy and Safety in Newly Diagnosed Chronic Myeloid Leukemia (BELA) trial of front-line bosutinib (for which the drug has not received FDA approval), diarrhea (any grade) was observed in 68% of patients (grade 3/4, 11%; Cortes et al., 2012). Diarrhea was typically mild or moderate in severity and occurred most commonly during the first 2 to 4 weeks on bosutinib (Cortes et al., 2011; Pfizer, 2013a).

Other GI events, including vomiting (reported in 32% of patients by 1 year minimum follow-up; grade 3/4 in 3%) and nausea (31% of patients; grade 3/4 in 1%), were also common in patients receiving front-line bosutinib (Cortes et al., 2012). Because bosutinib is a relatively new option for patients with CML, the long-term effects of bosutinib exposure remain unknown.

The 2014 NCCN guidelines recommend taking bosutinib with food and water to avoid GI symptoms (NCCN, 2014). Diphenoxylate/atropine or loperamide can effectively control diarrhea for most patients (Cortes et al., 2012). In patients with grade 3/4 diarrhea, bosutinib treatment should be interrupted until symptoms resolve to grade ≤ 1, at which time a reduced dose (400 mg once daily) is recommended (NCCN, 2014). Starting bosutinib-naive patients at 400 mg daily, with an increase to 500 mg daily after 2 weeks if the patient has not had loose stools, may reduce the frequency of diarrhea.

Liver enzyme elevations can also occur on bosutinib. With 1-year minimum follow-up in BELA, 22% of patients experienced grade 3/4 alanine aminotransferase (ALT) elevations and 11% experienced grade 3/4 aspartate aminotransferase (AST) elevations. More than half of these patients required dose interruptions and/or reductions and 71% experienced more than 1 elevation in ALT/AST levels (Cortes et al., 2012). Similar to GI events, liver-related AEs may be most common early during treatment. All patients should undergo hepatic enzyme tests monthly for the first 3 months and as clinically indicated thereafter. Patients with previous ALT/AST elevations should undergo more frequent monitoring (Pfizer, 2013a).

**Ponatinib**

Based on early data from the phase II Ponatinib Ph+ ALL and CML Evaluation (PACE) study, the FDA granted ponatinib accelerated approval in 2012 for the treatment of adult patients with CML or Ph+ acute lymphoblastic leukemia who were resistant to or intolerant of prior TKI therapy. However, subsequent analyses revealed increased arterial thrombotic events in patients receiving ponatinib, and the subsequent revised approval was narrowed to include only those patients with the T315I mutation or for whom no other TKI therapy is indicated, with further safety information included in the label (Ariad Pharmaceuticals, 2014). The recommended dose of ponatinib is 45 mg once daily with or without food, but the label cautions that an optimal dosage has not been identified, and the majority of patients treated with ponatinib have required dose reductions to 30 or 15 mg daily. The ponatinib label includes a boxed warning for vascular occlusion, heart failure, and hepatotoxicity; however, no contraindications are listed. Ponatinib is the most recently approved TKI, and like bosutinib, long-term clinical experience with ponatinib remains limited.

To date, arterial and venous thrombosis and occlusive events are the most clinically relevant AEs associated with ponatinib. Based on phase I data and PACE (with 2 years of follow-up), the ponatinib label reports that at least 27% of patients receiving ponatinib had these events (Ariad Pharmaceuticals, 2014). These included cardiac vascular occlusion in 12% of patients (including coronary artery occlusion and myocardial infarction, sometimes preceding or concurrent with heart failure), cerebrovascular occlusion (6%), peripheral arterial occlusive events (8%), and venous thromboembolic events (5%). Notably, vascular occlusive events occurred in 12% of patients aged < 50 years and in 16% of patients without known cardiovascular risk factors treated with ponatinib (Ariad Pharmaceuticals, 2014). Heart failure or left ventricular dysfunction was also reported in 8% of patients receiving ponatinib, including fatal and serious events in 5% of patients (Ariad Pharmaceuticals, 2014). Patients on ponatinib should be monitored for evidence of thromboembolism, vascular occlusion, and cardiac failure, and treatment should be interrupted or stopped if evidence of vascular occlusion or heart failure is detected (Ariad Pharmaceuticals, 2014).

Treatment-emergent hypertension is also common in patients receiving ponatinib. In PACE, 68% of patients with CML-CP experienced hypertension of any grade (39% had grade 3/4), and serious symptomatic hypertension occurred in 2% of the entire study population (Ariad Pharmaceuticals, 2014). Patients should be monitored for hypertension and receive therapies to normalize blood pressure when necessary. Ponatinib is recommended to be interrupted, dose reduced, or discontinued in patients with uncontrolled hypertension (Ariad Pharmaceuticals, 2014).

Reported as a dose-limiting toxicity in the ponatinib dose-escalation study, pancreatitis was also the most common serious AE in PACE (Cortes et al., 2012, 2013). By 12 months’ minimum follow-up in PACE, 7% of patients with CML-CP experienced treatment-related pancreatitis, including grade 3/4 pancreatitis (6%); lipase elevation (with or without pancreatitis) occurred in 21% of patients (Cortes et al., 2013). The ponatinib label recommends monitoring serum lipase levels at least biweekly for the first 2 months and monthly thereafter, with additional monitoring considered for cases of alcohol abuse or past pancreatitis. Ponatinib should be interrupted or discontinued in patients with pancreatitis or elevated lipase levels and not restarted until symptoms have completely resolved and lipase levels are below 1.5 times the upper limit of normal (Ariad Pharmaceuticals, 2014).

Although pancreatitis is most frequently observed early during treatment (Cortes et al., 2012), other AEs, such as fatigue, may become more clinically relevant long term. Among patients with CML-CP in PACE, 39% experienced fatigue or asthenia (any grade; grade 3/4, 3%) by 337 days’ median follow-up (Ariad Pharmaceuticals, 2014). Patients with fatigue on ponatinib can be managed similarly to those with fatigue on the other TKIs.

## CONSIDERATIONS FOR PRACTICAL LONG-TERM MANAGEMENT

Additional differences between the various TKIs inform the clinical management of CML. The concept of treatment-free remission has shown preliminary feasibility, and patients who achieve sustained, deep molecular responses may eventually have the option to cease all TKI therapy. Treatment discontinuation would also eliminate any chronic AEs related to ongoing TKI exposure (Ross et al., 2013); thus, selecting a TKI capable of inducing deep, sustained molecular responses may relate to AE management. Of note, treatment-free remission is investigational and should be attempted only in the context of a clinical trial with physician supervision and strict monitoring per protocol.

The relative cost of each TKI may also impact treatment decisions in some cases. Recently, a number of CML experts publicly suggested that the cost of a lifetime of TKI therapy may be unsustainable (Experts in Chronic Myeloid Leukemia, 2013). Once available, generic drugs are expected to reduce this cost dramatically. It remains to be seen what effects expiration of the US patent on imatinib in 2015 and the availability of generic imatinib will have on TKI prescribing decisions, especially in light of the growing body of data demonstrating superior efficacy of newer TKIs vs imatinib (Jabbour et al., 2014; Larson et al., 2014).

Enabling patients to achieve optimal levels of response and maximizing their long-term prognosis remain the priorities of CML therapy. Although each of the available TKIs affords some level of response in most patients, the ability to induce optimal responses and prevent disease progression can differ markedly between TKIs. Whereas dasatinib, nilotinib, and bosutinib each induced significantly faster achievement of major molecular remission compared with imatinib in newly diagnosed patients, only nilotinib significantly reduced the risk of progression to AP/BC compared with imatinib (Cortes et al., 2012; Kantarjian et al., 2010; Saglio et al., 2010).

The ability to identify responders at baseline or early during treatment is highly clinically relevant. Achievement of an early molecular response (ie, *BCR-ABL mRNA* transcript levels ≤ 10% on the International Scale [BCR-ABL^IS^] within 3 or 6 months) is associated with improved long-term outcomes. Several recent studies demonstrated that patients with BCR-ABL^IS^ > 10% at 3 or 6 months on front-line imatinib had poorer long-term outcomes than those with BCR-ABL^IS^ ≤ 10% at 3 or 6 months (Hanfstein et al., 2012; Hughes et al., 2010; Marin et al., 2012; Quintas-Cardama et al., 2009). Patients in ENESTnd with BCR-ABL^IS^ ≤ 10% at 3 months on nilotinib 300 mg twice daily had significantly improved 4-year progression-free survival vs patients with BCR-ABL^IS^ > 10% at 3 months (95.2% vs 82.9%, respectively; *p* = .0061) (Hughes et al., 2014). Similar trends have been reported with front-line dasatinib and imatinib (Hughes et al., 2014; Jabbour et al., 2014; Jain et al., 2013; Marin et al., 2012) and second-line nilotinib (Branford et al., 2012). Importantly, fewer patients achieve an early molecular response on imatinib than on either dasatinib or nilotinib (Hughes et al., 2014; Jabbour et al., 2014).

Achievement of early molecular responses was recently incorporated into CML treatment guidelines (Table 3; Baccarani et al., 2013; NCCN, 2014) and should be considered along with toxicities, cost, and treatment goals when decisions are made about the treatment of patients with CML.

**Table 3:**
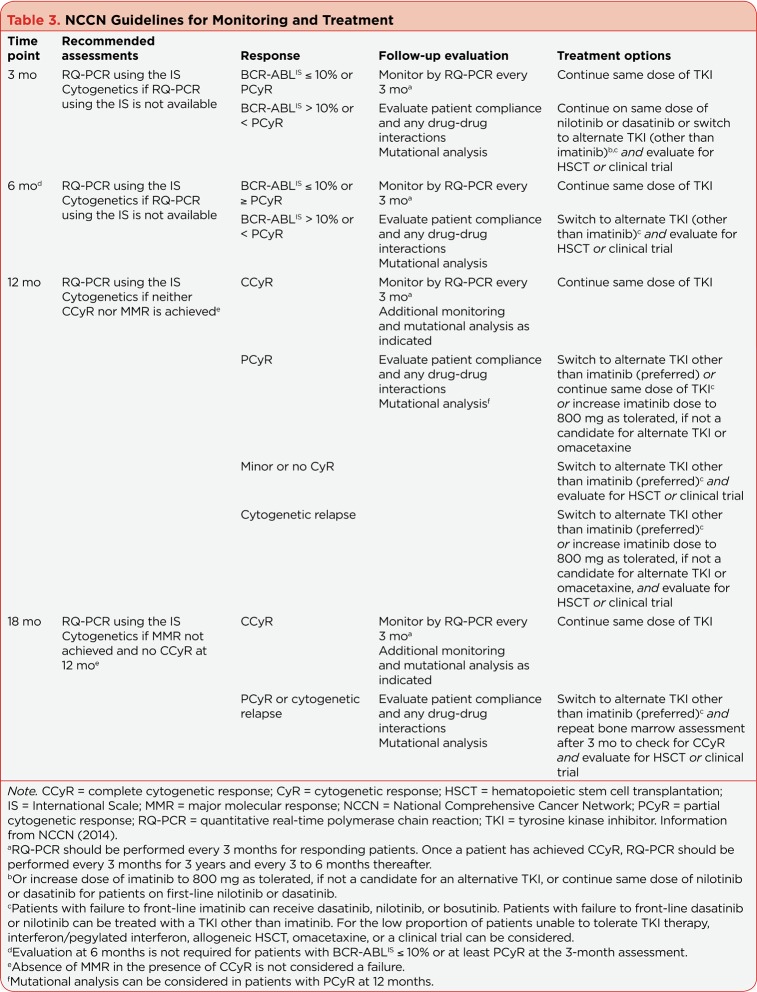
NCCN Guidelines for Monitoring and Treatment

## IMPLICATIONS FOR ADVANCED PRACTITIONERS

Because several approved BCR-ABL–targeted TKIs are now available for the treatment of CML-CP, treatment and management decisions for HCPs are becoming more complex. Current recommendations are that responding patients remain on TKI therapy indefinitely (NCCN, 2014), and treatment decisions should consider the potential long-term effects for each TKI from the time of initial diagnosis.

Establishment of objective guidelines to facilitate treatment decisions is limited by a relative dearth of long-term data regarding AEs associated with each TKI. Furthermore, some AEs (e.g., cardiovascular complications) are observed with multiple TKIs; additional information regarding the relative severity and mechanisms of these events will aid decision making in the future. As long-term data accumulate, useful objective criteria may become evident.

Improving the management of relevant comorbidities is another important goal. Integrated management of various comorbidities might dampen their impact and relieve many TKI-associated AEs. Improved holistic patient care could involve either well-integrated communication among a team of HCPs and specialists (multidisciplinary approach) or a broadened role for a single treating HCP who has been entrusted with managing not only a patient’s CML but also relevant comorbid conditions.

Achieving a rapid and deep response against the CML itself is only the first step in the clinical management of Ph+ CML; thereafter, advanced practitioners must continue to manage AEs over the years or even decades of ongoing TKI therapy. Proper adherence to TKI therapy is significantly associated with achievement of molecular responses, including deeper molecular responses, whereas lower adherence is associated with greater symptom severity and reduced quality of life (Efficace et al., 2014; Marin et al., 2010). Thus, by encouraging education and communication between patients and their advanced practitioners, there is an opportunity to improve adherence and achieve optimal long-term monitoring and management of CML. The availability of several effective therapeutic options allows caregivers and patients to make treatment decisions tailored to each individual’s circumstances.

**Acknowledgments**

Financial support for medical editorial assistance was provided by Novarti Pharmaceuticals. The authors thank Staci Heise, PhD, and Karen Kaluza, PhD (Articulate Science), for medical editorial assistance with this manuscript.
